# Can molecular projected density of states (PDOS) be systematically used in electronic conductance analysis?

**DOI:** 10.3762/bjnano.6.128

**Published:** 2015-06-02

**Authors:** Tonatiuh Rangel, Gian-Marco Rignanese, Valerio Olevano

**Affiliations:** 1 Institute of Condensed Matter and Nanosciences, Université catholique de Louvain, Chemin des Étoiles 8, bte L7.03.01, 1348 Louvain-la-Neuve, Belgium; 2 European Theoretical Spectroscopy Facility (ETSF); 3 Present address: Molecular Foundry, Lawrence Berkeley National Laboratory, Berkeley, California 94720, USA; 4 CNRS, Institut Néel, F-38042 Grenoble, France; 5 University Grenoble Alpes, F-38000 Grenoble, France

**Keywords:** benzene-diamine, benzene-dithiol, DFT-Landauer, molecular electronics, nanoelectronics, quantum transport

## Abstract

Using benzenediamine and benzenedithiol molecular junctions as benchmarks, we investigate the widespread analysis of the quantum transport conductance in terms of the projected density of states (PDOS) onto molecular orbitals (MOs). We first consider two different methods for identifying the relevant MOs: (1) diagonalization of the Hamiltonian of the isolated molecule and (2) diagonalization of a submatrix of the junction Hamiltonian constructed by considering only basis elements localized on the molecule. We find that these two methods can lead to substantially different MOs and hence PDOS. Furthermore, within Method 1, the PDOS can differ depending on the isolated molecule chosen to represent the molecular junction (e.g., with or without dangling bonds); within Method 2, the PDOS depends on the chosen basis set. We show that these differences can be critical when the PDOS is used to provide a physical interpretation of the conductance (especially when its value is small, as it happens typically at zero bias). In this work, we propose a new approach in an attempt to reconcile the two traditional methods. Although some improvements were achieved, the main problems remain unsolved. Our results raise more general questions and doubts on a PDOS-based analysis of the conductance.

## Introduction

According to Moore’s law, in a decade or so, the downscaling of conventional silicon-based electronics will achieve its ultimate nanoscale limit. Molecular electronics, or electronics at the nanoscale, is considered one of the most difficult technological challenges. The construction, measurement and understanding of electronic devices constituted of single molecules between metal electrodes is currently a major concern of fundamental research. Today, different techniques are available to realize molecular junctions in laboratories, such as electromigration methods, mechanical strain and scanning tunneling microscopy to open small gaps between gold leads that can host (with a small but non-negligible probability) single molecules from a wetting solution [1–3]. The complete characterization of such junctions (including the measurement of their current–voltage characteristics) is, however, still difficult to achieve. In order to obtain a reliable single-molecule zero-bias conductance, it was suggested to resort to a statistically significant sample of tens of thousands of measurements [4]. From this breakthrough work, it is now possible to quote the zero-bias conductance of some molecular junctions such as benzene-diamine (BDA) and benzene-dithiol (BDT) between gold leads. Nevertheless, important characterization uncertainties still persist. For instance, in these experiments, the junction geometry is not measured and hence is unknown. Given these difficulties, resorting to theory could reveal a valid approach to understand and interpret the experimental observations. The theoretical description of the electronic quantum transport in molecular junctions or nanostructures relies on established frameworks [5–6] like the Kubo–Greenwood [7–8] or the Landauer [9] formalisms, or the non-equilibrium Green’s function theory [10–12]. In the last two decades, the combination of these formalisms with density functional theory (DFT) or many-body perturbation (MBPT) theory allowed for the establishment of ab initio approaches to quantum transport. The DFT-Landauer framework is one of the most popular. It has proven successful in calculating zero-bias conductances in good agreement with the experiment in some systems such as the hydrogen molecule between platinum wires [13]. In other systems, such as organic molecule junctions, the DFT-Landauer estimate can be several orders of magnitude larger than the experiment [1,14]. Several solutions have been proposed to alleviate this discrepancy such as: self-interaction corrections [15–16], hybrid mixed Hartree–Fock approaches [17], a many-body model [18–21] or ab initio GW corrections [22–23], arising a yet-to-be solved controversy [24–32]. In addition to calculations and measurements, a physical interpretation of the conductance is needed. In the end, a complete picture of the mechanisms governing quantum transport is needed in order to fully understand the behavior of the molecular junction as an electronic device. Thus, it is important to establish a relationship between the conductance and the electronic structure, for example, by determining the main constituents influencing the absolute value of the zero-bias conductance. A very common approach for providing such an interpretation proceeds as follows. A set of molecular orbitals (MOs) associated to the central molecule are identified and classified according to the energy levels (e.g., the highest occupied molecular orbital (HOMO), or the lowest unoccupied molecular orbital (LUMO), or the next LUMO (LUMO+1), etc.). Then, the total electronic density of states (DOS) is decomposed into the projected density of states (PDOS) associated with each different MO. Finally, by directly comparing the conductance profile, 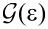, with the various PDOS, one tries to establish a correspondence between conductance features and MOs. In particular, it is attempted to understand which MO has the largest influence on the zero-bias conductance. The purpose of this work is to investigate how meaningful (or on the contrary, misleading) this analysis is. How reliable are the resulting interpretations? How pertinent is it to a correct understanding of the behavior of the system? We analyze two common benchmarks, the above mentioned molecular junctions of BDA and BDT between gold leads, in order to answer these questions and to solve the problems evidenced in traditional methodologies. In particular, we propose a new method to identify MOs and the associated PDOS, which clearly contributes to this goal, although further work is still required. Although the findings of this work may seem quite theoretical at first sight, they will have an important impact in the experimental community. Indeed, the theoretical analysis of quantum transport is often used for interpretation of the measurements by predicting trends (for example, for the sign of the thermopower), for obtaining independent arguments, or for checking the validity of the experimental work.

This paper is organized as follows: The first section introduces quantum transport ab initio theory, together with the definitions of all the relevant quantities and the two traditional methods to identify MOs and PDOS. In the second and third sections, we present the results for the BDA and BDT molecular junctions, respectively. The forth section is devoted to the presentation of our new method and the results when applied to BDT. The last section gives a critical discussion of the physical meaning of the interpretation provided by the traditional methods and our new one.

## Theory

In the DFT-Landauer framework, the molecular junction is modeled by a central region (C) connected to two semi-infinite leads (left (L) and right (R)). Its conductance as a function of the energy of the injected electrons, 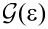, is given by the Landauer formula:


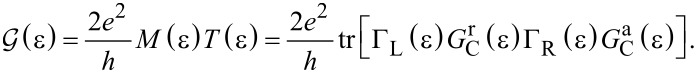


*M*(ε) is the number of modes at a given energy, ε, and *T*(ε) is their transmittance. Γ_L/R_(ε) is the left/right-lead injection rate. 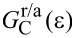 is the retarded/advanced Green function for the central region. The quantities 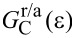 and Γ_L/R_(ε) can be obtained from the DFT electronic structure (i.e., the energies ε*_n_* and wavefunctions 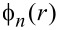) of the central region containing an “extended molecule” and of the leads (treated as infinite, periodic solids), respectively. The central extended molecule consists of the molecule itself plus some layers belonging to the leads. The number of included layers (typically 3 or 4) should account for the relaxation of both the atomic and the electronic structures of the junction. The value assumed by 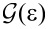 at the Fermi energy ε_F_ (which will be set to 0 in the following, 
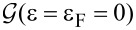
) is an observable that can be directly measured in experiments and is referred to as the zero-bias conductance. The junction conductance depends on the nature and the shape of the leads, the geometric/atomic structure of the molecule–lead contact, and the molecule itself. Experiments and calculations very often only consider gold for the leads, so that these parameters can be considered as a constant. In contrast, the geometry of the molecule–lead contact may vary quite a lot, but in many cases, it is not known, and furthermore, it cannot be experimentally controlled. In practice, experiments only measure conductance averaged over the different possible geometries. In the end, the main factor influencing the junction conductance is the central molecule. Therefore, there is significant interest in how the conductance changes with varying chemical composition or with the atomic structure of the central molecule. Furthermore, when looking at the overall representation of the molecular junction, the central molecule appears as a “bottleneck” in the stream of electrons flowing from one lead to the other. For this reason, it is believed that the molecule itself and its electronic structure has a deep influence on the conductance. The interpretation of the conductance profile, 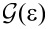, or of the zero-bias conductance, 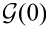, is often carried out by referring to the projected density of states onto molecular orbitals (see next section). Traditionally, these are identified using the two methods that will be described in detail later.

### Interpretation of the conductance by the PDOS

Supposing that a set {*m*} of molecular orbitals with corresponding wavefunctions 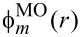 have been identified, the projected density of states, ρ*_m_*(ε), on the molecular orbital *m* is defined as

[1]
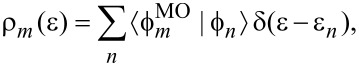


where *n* runs over all the states of the central extended molecule with wavefunction 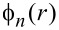 and energy ε*_n_*. In Equation 1, the Dirac delta function is usually replaced by a Gaussian function with a given broadening. As discussed above, 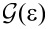 is primarily determined by the electronic structure of the central extended molecule. In particular, the DOS, 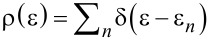, should play a major role. For instance, the conductance will be zero when the number of modes *M*(ε) = 0, and so will be the density of states. Hence, it is quite natural to interpret the conductance with the help of the DOS. More specifically, it has become very common to analyze 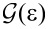 in terms of the different partial molecular components that enter the full DOS, that is, the PDOS on the various MOs [33–37]. Since the energy region of interest for the conductance is around the Fermi level, one usually takes into account the molecular orbitals around the fundamental gap, e.g., the highest occupied molecular orbital (HOMO), the lowest unoccupied molecular orbital (LUMO), and their successors and predecessors, LUMO+1, LUMO+2, HOMO−1, etc. The analysis of the conductance in terms of the PDOS is based on a one-to-one comparison of 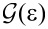 with ρ*_m_*(ε) for some chosen MOs. Whenever a peak in 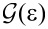 coincides with a peak in a ρ*_m_*(ε), that molecular orbital, *m*, is said to “drive” the peak of conductance. The specific case of the zero-bias conductance is a bit particular. Indeed, very often, 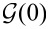 is quite small and the main conductance peaks are several eV away. The zero-bias conductance is actually interpreted as the tail of one of these peaks. However, there is some ambiguity regarding which MO will be said to drive 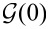. Indeed, it can be chosen as:

- the MO corresponding to the peak closest to the Fermi level (ε = 0) [38–39], or
- the MO presenting the highest PDOS value at ε = 0, no matter how far the PDOS maximum is from ε = 0 [23,40–41].

### Identification of the molecular orbitals

The molecular orbitals, 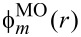, are the fundamental ingredients of the PDOS (see Equation 1). As shown below, the approach chosen for identifying the MOs strongly affects the PDOS and the consequent interpretation of the conductance spectrum. Two main methods have been used so far in the literature for identifying MOs.

- Method 1: The 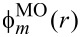 are chosen to be the eigenfunctions of the Hamiltonians of the uncontacted, gas phase, isolated molecule [41]. For consistency, they are usually determined using exactly the same supercell of the extended molecule, as in the molecular junction calculation, and by removing the atoms of the leads.
- Method 2: The Hamiltonian of the extended molecule is first expressed on a real-space localized basis set. This can be achieved, for instance, using maximally localized Wannier functions (MLWFs) [42]. The 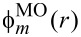 are then chosen as the eigenfunctions of the submatrix constructed by considering only basis elements localized on the molecule [13].

There is no obvious reason why the MOs identified using these two different procedures should coincide. Furthermore, it is not evident which method is preferred with respect to the assumed purpose, that is, the analysis of the conductance. Method 1 coincides with the rigorous definition of MOs from a chemistry perspective for the isolated molecule. However, the electronic structure of the extended molecule (taking into account charge transfer and other modifications induced by the contact between the molecule and the leads) is clearly much more important with respect to the conductance profile. Thus, Method 2 appears more relevant for the analysis of the conductance. Note that choosing one of these methods does not affect the conductance profile provided that convergence is reached. What actually changes is rather the PDOS, and hence, the interpretation of the conductance in these terms.

### Computational details

Our calculations are carried out within the DFT-Landauer framework. The exchange-correlation energy is approximated using the PBE functional [43]. We use ABINIT [44] for ground state calculations and WanT [45–46] to construct Wannier functions and for conductance calculations. All the results presented here are obtained by well-converged calculations, using the same convergence parameters as in [23], which are consistent and in agreement with the literature.

## Results

### Benzene-diamine

#### BDA molecular orbitals

**Figure 1:**
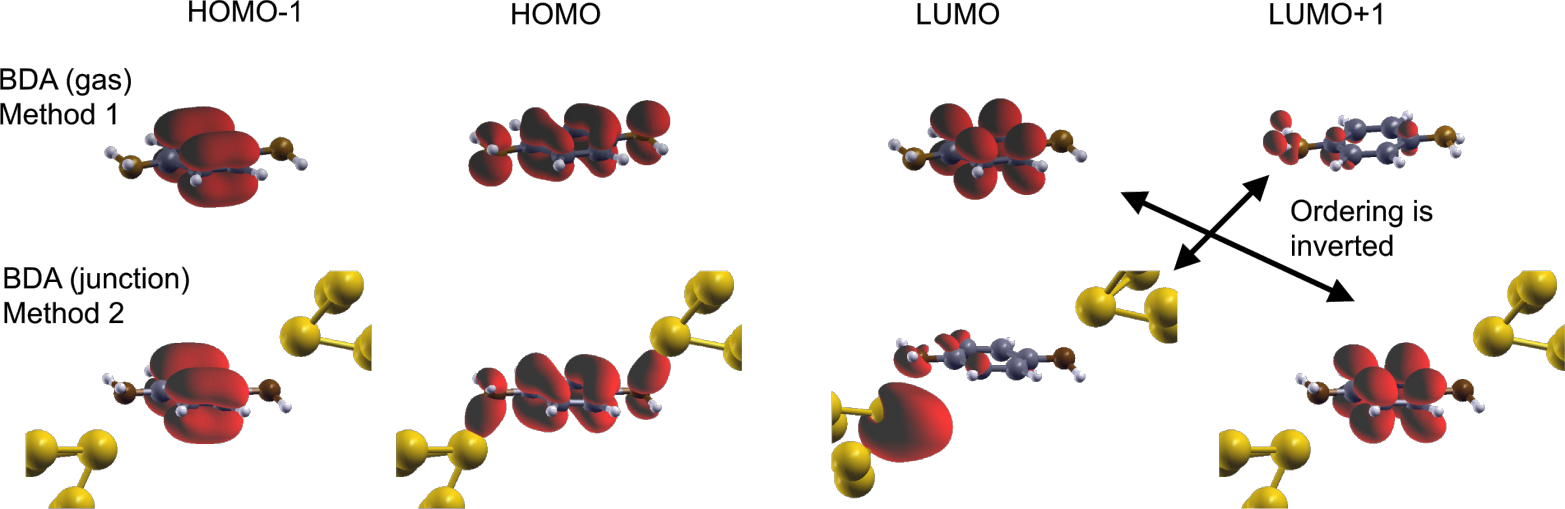
Electronic density isosurfaces (red) of the HOMO−1, HOMO, LUMO and LUMO+1 molecular orbitals of BDA as obtained with the two traditional methods (see text). The ordering of the LUMO and LUMO+1 is inverted in the two methods. The localized MOs (HOMO−1 and LUMO (gas) or LUMO+1 (junction)) look remarkably similar for both methods. In contrast, the HOMO and LUMO in the junction present a clear bonding with the leads and thus slightly differ from the corresponding MOs in gas phase. Hydrogen, carbon and sulfur atoms are represented by white, grey and green spheres, respectively.

In Figure 1, we show the molecular orbitals of BDA calculated with Methods 1 and 2. They are analogous to previously found MOs [41]. While the HOMO−1 molecular orbitals are very similar, the HOMO show non-negligible differences: the bonding character with the leads is more important when using Method 2, as indicated by the more pronounced lobes on the N atoms that point towards the gold adatoms.

We observe a close similarity between the LUMO from Method 1 and the LUMO+1 from Method 2, as if there were a change in the ordering of the corresponding eigenvalues between the two methods. Notice that the energy difference between the LUMO and the LUMO+1 is ≈0.5 eV, which is enough to exclude their degeneracy. In contrast, the LUMO+1 from Method 1 resembles the LUMO from Method 2 but with some small differences: the bonding character with the leads is again more pronounced when using Method 2. In fact, the corresponding density arises from a MLWF basis element, which is localized on the gold–amino bond and not clearly identifiable as purely belonging to gold or to the molecule. In this MO, important differences are also found for the lobes on the benzene ring: in Method 1, the lobes are mainly on the opposite C atoms along the molecule long axis; whereas, in Method 2, they are on the C atoms close to the Au adatom. These differences will induce non-negligible differences in the PDOS analysis, as will be shown in the next section.

#### PDOS and interpretation of the conductance

**Figure 2:**
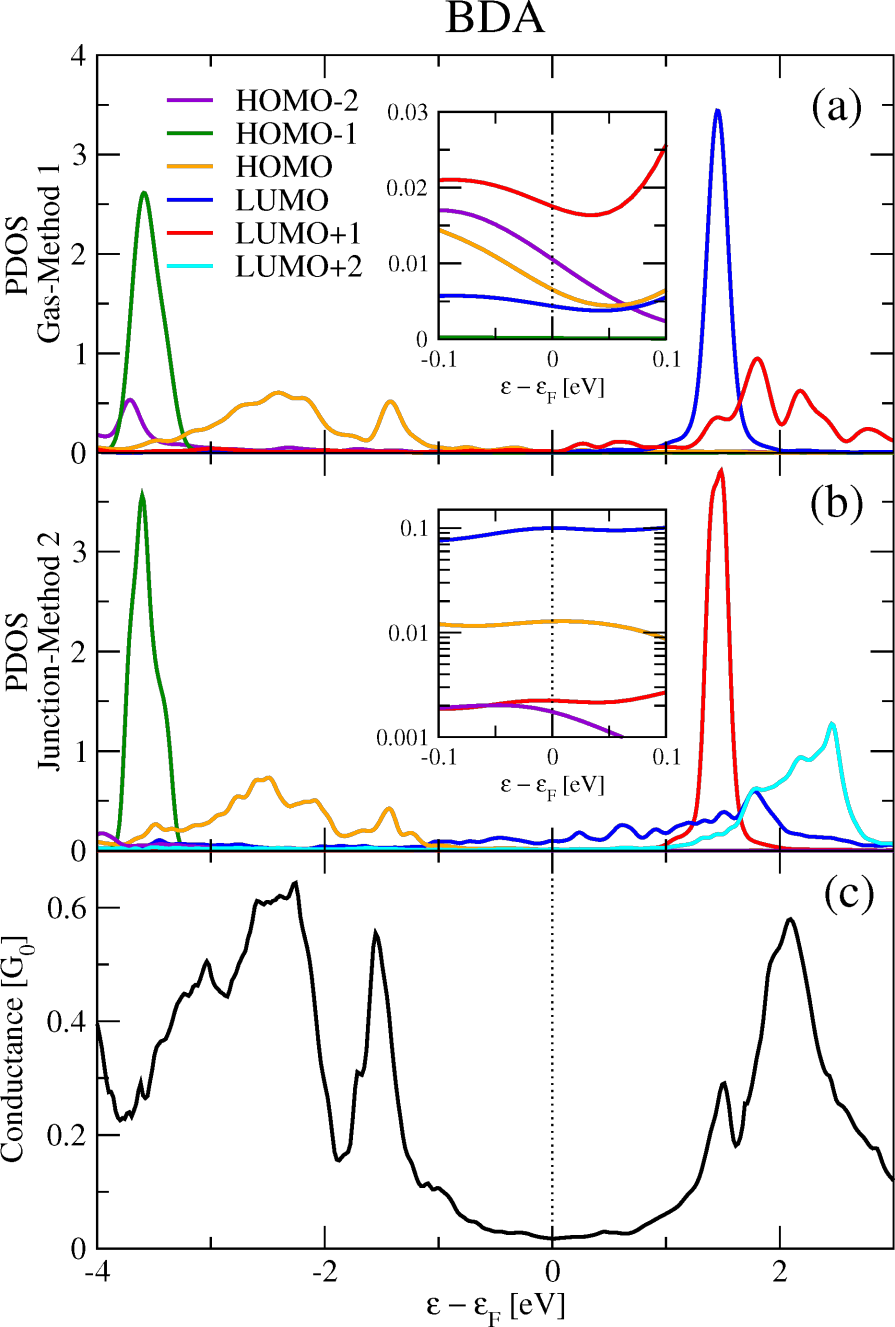
Projected density of states (a,b) and conductance (c) of benzene-diamine (BDA). The PDOS for the different molecular orbitals (from HOMO−2 to LUMO+1) have been obtained with (a) Method 1 and (b) Method 2 (see text). The insets show a zoom on the PDOS around the Fermi energy region. Notice that in the inset of (b), the PDOS is presented in logarithmic scale.

In Figure 2c, we show the conductance of BDA calculated in the Landauer-DFT framework using the PBE approximation. As it is usually done in literature, when providing a physical interpretation of the conductance, we also present the PDOS as calculated using Method 1 (Figure 2a) and Method 2 (Figure 2b). The position and height of the main features are in very good agreement with previous work [41].

**Figure 3:**
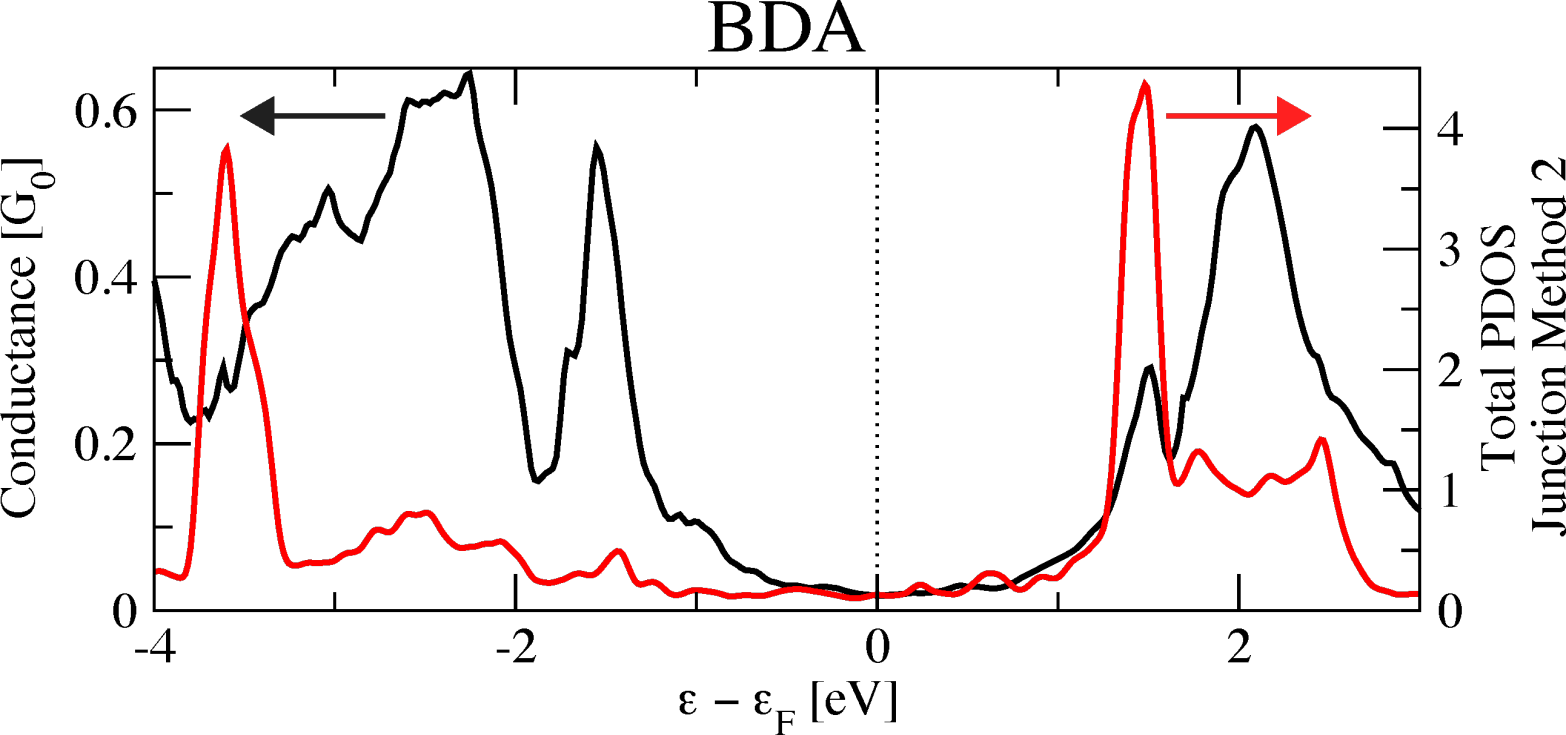
Total molecular PDOS (red line) and conductance (black line) of BDA. The total molecular PDOS is the sum of the PDOS onto the MOs from HOMO−2 to LUMO+2 as obtained from Method 2.

The two PDOS look quite similar but with differences that can be associated to the already discussed discrepancies between MOs. In particular, we observe the change in the ordering between the LUMO and the LUMO+1 from Method 1 to 2. The PDOS onto non-hybridized MOs (HOMO−1 and LUMO/LUMO+1 in Method 1/2) look similar, whereas the PDOS onto the HOMO and LUMO+1/LUMO in Method 1/2 present differences, as expected from the MO plots. Finally, the PDOS onto HOMO−2 seems to have more weight in Method 1 than in Method 2. When interpreting the conductance profile, one can associate the small conductance peak at ≈1.5 eV with the intense LUMO and LUMO+1 PDOS peaks observed in Methods 1 and 2, respectively. The conductance structure arising at energies *>*0 eV with maximum at 2 eV could be correlated to the other unoccupied molecular orbital (LUMO+1 of BDA gas otherwise LUMO of BDA junction), as well as the LUMO+2. The peak in the conductance at approx. −1.5 eV could be related to the HOMO PDOS peak at approx. −1.4 eV, and so also the structure from −2 down to −3.8 eV. The HOMO−1 and its PDOS peak at approx. −3.6 eV is not reflected in the conductance. However, when performing a one-to-one comparison of the conductance with the total PDOS on the various MOs (Figure 3), the relationship is weak, even qualitatively.

An interpretation of the 0-bias conductance will now be discussed. Following one possible interpretation scheme very common in the literature, the zero-bias conductance appears in the tail of the conductance peak at −1.5 eV (HOMO), although the smallest peak at +1.5 eV (associated with the PDOS onto the LUMO/LUMO+1 from Method 1/2) is equally distant. According to this interpretation, the zero-bias conductance is driven by the HOMO, although a contribution from the LUMO from Method 1 (alias the LUMO+1 from Method 2) is expected. These conclusions are contrasted by another approach which rather looks at the absolute values of the PDOS at the Fermi energy (see Figure 2 insets showing zooms on the Fermi energy regions). According to this scheme, the other unoccupied MO (the LUMO+1 from Method 1, alias the LUMO from Method 2) drives the zero-bias conductance. In fact, both methods agree on the fact that this MO (labeled differently) presents the largest PDOS value at the Fermi energy. Nevertheless, its corresponding PDOS value at 0 eV disagrees by one order of magnitude: from 0.1 in Method 2 and 0.02 in Method 1. The next MO presenting an important PDOS value at the Fermi energy is the HOMO−2 from Method 1, with a value not much smaller than the LUMO+1, implying that the HOMO−2 has a certain weight on the zero-bias conductance. However, this is the HOMO from Method 2 with a marked gap (from 0.1 to 0.01). Both methods agree about the HOMO PDOS absolute value (≈0.01) at ε = 0, probably by mere coincidence given the disagreements mentioned above. Summarizing, when interpreting the BDA zero-bias conductance, we are confronted with three problems: (1) the arbitrariness in the labeling of MOs (the LUMO in Method 1 becomes the LUMO+1 in Method 2, and vice versa), (2) the dependence on the method to identify the MOs, and (3) the dependence on the interpreting approach. Hence the PDOS analysis of 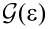 is affected by some ambiguity.

### Benzene-dithiol

#### BDT molecular orbitals

**Figure 4:**
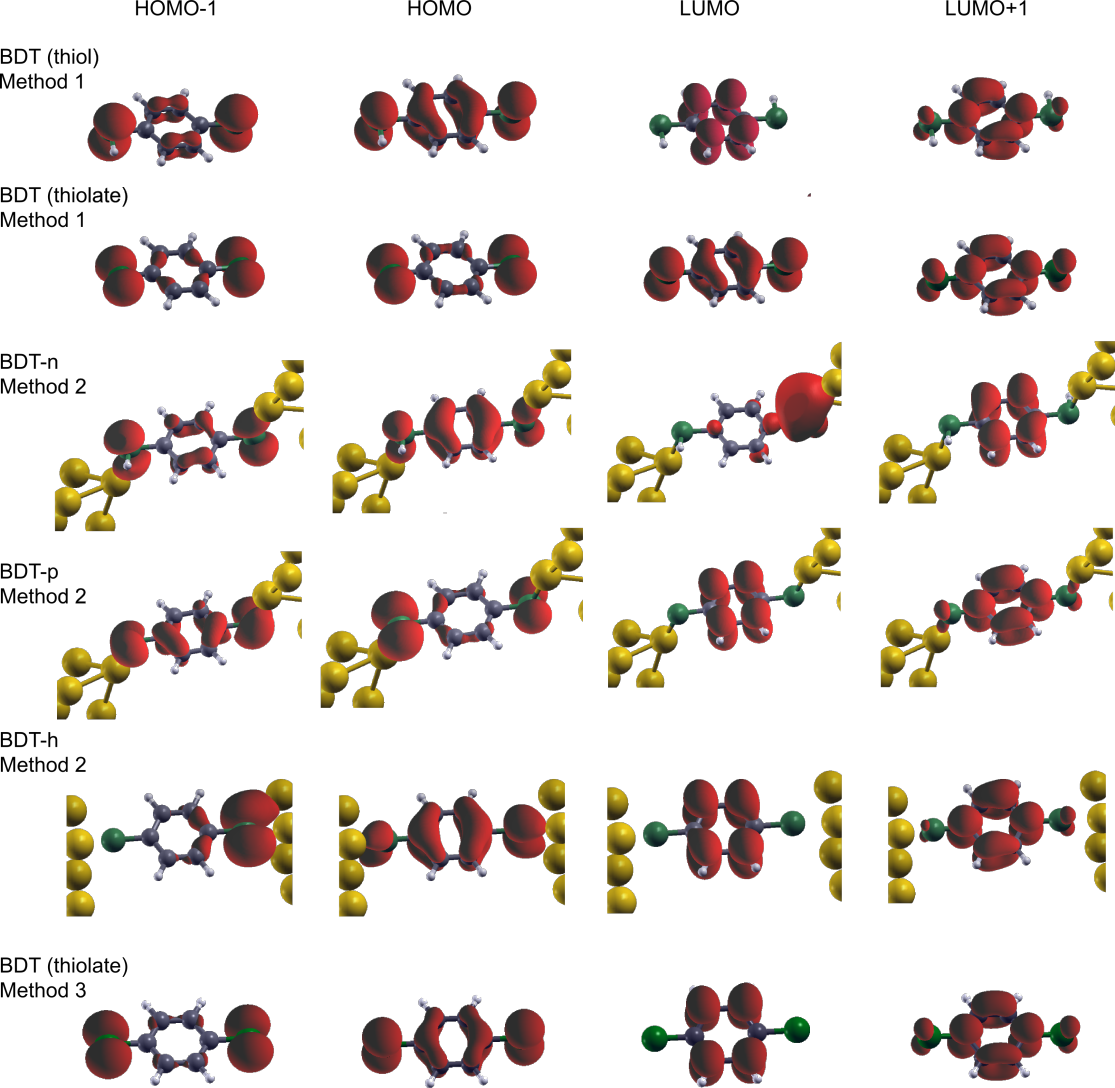
Electronic density isosurfaces (red) of the HOMO−1, HOMO, LUMO and LUMO+1 molecular orbitals of BDT as obtained with the two traditional methods as well as with the new method (see text). For Method 1, the dithiol and dithiolate molecules are considered. For Method 2, the different molecular junction geometries (BDT-n, BDT-p and BDT-h) are examined. For Method 3, a charge of +0.5*e*^−^ was added to the dithiolate molecule in order to account for the transfer of charge to the molecule from gold atoms in the BDT-h junction. The resulting orbitals are very similar to those obtained with Method 2 for BDT-h. Hydrogen, carbon and nitrogen atoms are represented by white, grey and brown spheres, respectively.

We now consider a more complex case: the benzene-dithiol (BDT)–gold junction. Experimental and theoretical works concluded that the BDT–gold junction can be stable in several different atomic structures/geometries [16,47–51]. To account for different hybridizations and bonding motifs, three geometries are studied here: the sulfur atom of the benzene-dithiol molecule can adsorb onto an extra gold adatom without losing the bound hydrogen atom (BDT-n); the benzene-dithiol molecule can lose the hydrogen atom, thus becoming benzene-dithiolate, and bind its sulfur atom to an extra gold adatom in a pyramidal structure (BDT-p); or the benzene-dithiolate can bind to three equidistant gold atoms in the hollow structure (BDT-h). These geometries are shown in Figure 4, where we show the MOs of BDT calculated with different methods.

For Method 2, we show the molecular orbitals obtained for the three different junction geometries: BDT-n, BDT-p and BDT-h. They are very similar to those previously obtained [13], especially given the differences in the considered geometries. In [52], an alternative set of MOs are shown for BDT-h, obtained within Method 2 by considering only the localized orbitals on the benzene molecule (excluding the S atoms). For Method 1, we depict both the cases of benzene-dithiol and benzene-dithiolate. The latter might better represent the BDT molecule in the BDT-p and BDT-h junctions where it loses a hydrogen atom before binding. However, this is not so straightforward. Besides the effective chemical composition of the molecule in the junction, other chemical/physical effects (e.g., saturation of bonds or transfer of charge), may be considered [24–27]. We start by analyzing the MOs from Method 1. The MOs for the dithiol and dithiolate molecules present a few similarities. The LUMO+1 are similar in shape. The HOMO of the dithiol molecule resembles to the LUMO of the dithiolate molecule, with an exchange of the ordering, as was seen in BDA (see the previous section). Nevertheless, other MOs strongly differ. Thus, the identification of MOs using Method 1 strongly depends on the molecule (dithiol vs dithiolate). We now present the analysis of the MOs obtained with Method 2. We focus on BDT-p, the junction in which the interpretation of conductance using the PDOS is the most critical of all the cases considered here, as will be seen later. The LUMO+1 from Method 2 looks very similar to the LUMO+1 from Method 1 for both the dithiol and dithiolate molecules, yet with differences on the sulfur atom. The LUMO from Method 2 corresponds to the LUMO from Method 1 for the dithiol molecule, but it does not correspond to any MO from Method 1 for the dithiolate molecule. On the other hand, the HOMO from Method 2 is similar to the HOMO from Method 1 for the dithiolate molecule, but it differs from all MOs from Method 1 for the dithiol molecule. Finally, the MOs which look more similar to the HOMO−1 from Method 2 are the HOMO from Method 1 for the dithiol molecule and the LUMO from Method 1 for the dithiolate molecule. From the above discussion, it appears that no one-to-one correspondence can be established between the MOs obtained with the two methods nor between the MOs from Method 1 both for the dithiol and dithiolate isolated molecules. The BDT-p MOs from Method 2 are halfway between the MOs from Method 1 for the dithiolate and dithiol molecules.

#### PDOS and interpretation of the conductance

**Figure 5:**
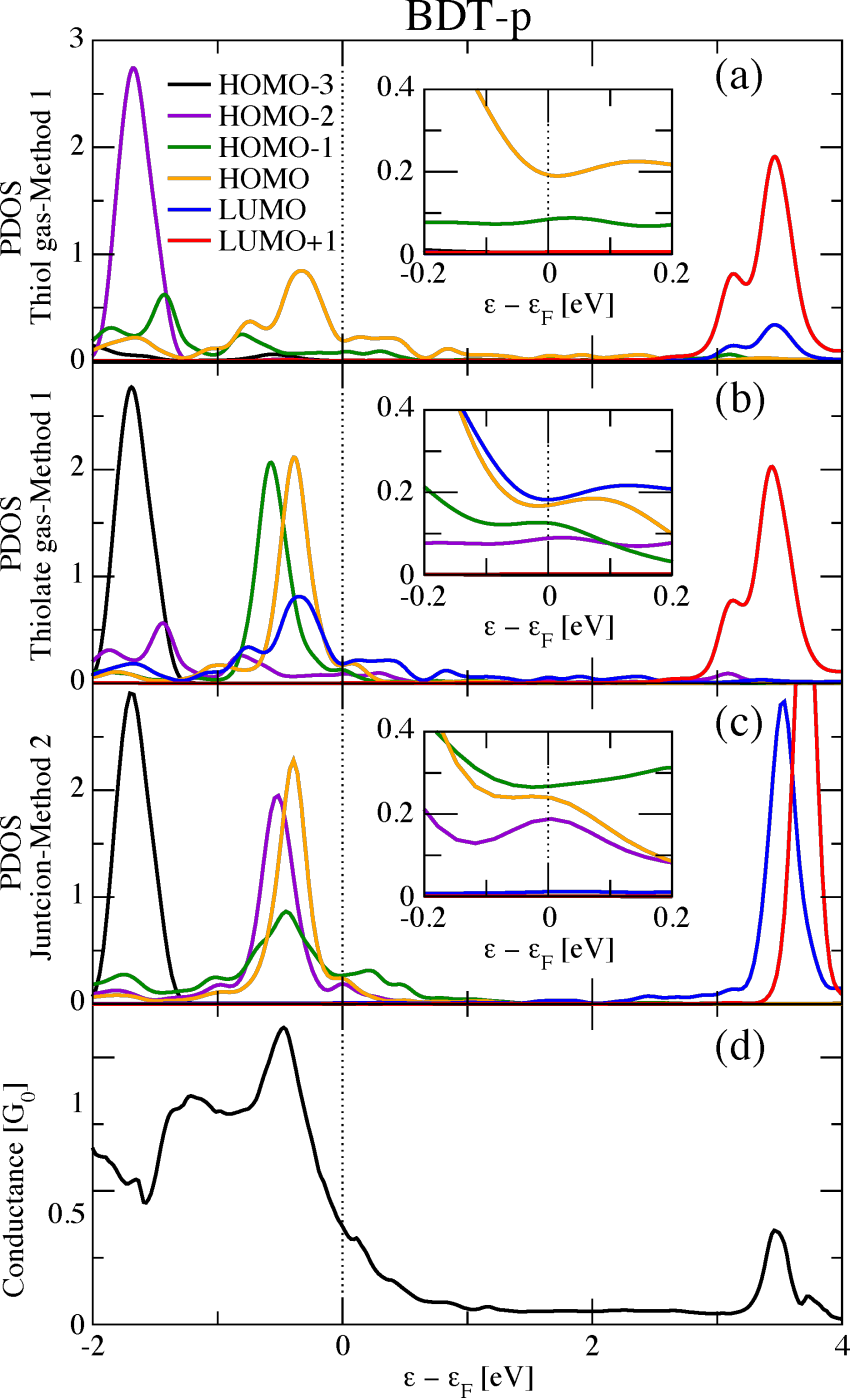
Projected density of states (a,b,c) and conductance (d) of benzene-dithiol in the pyramidal geometry (BDT-p). The PDOS for the different molecular orbitals (from HOMO−3 to LUMO+1) were obtained with (a) Method 1 based on the dithiol molecule, (b) Method 1 based on the dithiolate molecule, and (c) Method 2 (see text). The insets show details of the PDOS around the Fermi energy region.

We now move to the analysis of the most critical case among the examples investigated here regarding the interpretation of the conductance in terms of the PDOS: benzene-dithiol in the pyramidal geometry (BDT-p). In Figure 5 we present the Landauer-DFT conductance of BDT-p. Additionally, we present three different PDOS calculated following Method 1 (gas phase) and Method 2 (junction). For the former, both the dithiol and dithiolate molecules are considered.

Without entering into all details, it is clear that the PDOS strongly depends on the method used to calculate it, reflecting previously seen differences in the MOs. For instance, the zero-bias conductance seems dominated by the HOMO from Method 1 for the dithiol molecule, since the PDOS onto the HOMO is the closest to the Fermi level and it also provides the highest contribution at that level (see the inset), with a minor contribution from the HOMO−1. When using Method 1 for the dithiolate molecule, the zero-bias conductance seems equally driven by the HOMO and LUMO, with also some contribution from the HOMO−1 and the HOMO−2. Finally, using Method 2, the HOMO−1, the HOMO and the HOMO−2 (in decreasing order) are the most important contributions at zero-bias. Though some discrepancies can be ascribed to simple relabeling of the same MO, one cannot pass over more important differences among the methods. In conclusion, we could not find a rigorous definition of the MOs and associated PDOS for the BDT-p case when using the traditional methods. As a consequence, the PDOS interpretation of the conductance does not rely on stable grounds.

### New method for identifying molecular orbitals

#### Charged isolated molecules

**Figure 6:**
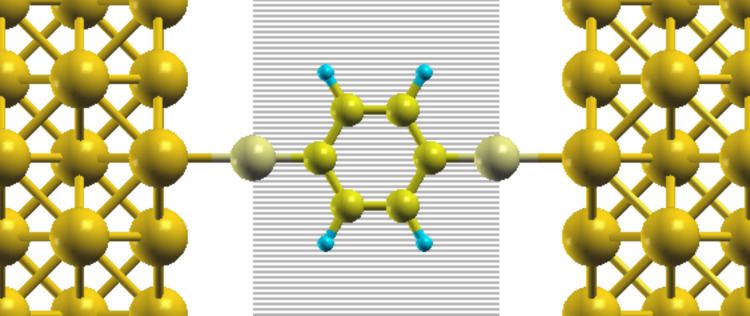
Scheme representing the integration volume (shadowed area passing through the two S atoms of the BDT-h junction) used for Method 3.

In order to reconcile the two main methods found in the literature (i.e., to reduce their differences and solve related difficulties), here we propose a new approach that is based on an evolution of Method 1. Method 3: The 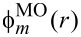 are chosen as the eigenfunctions of the Hamiltonian of the uncontacted, gas phase, isolated molecule, to which some charge is added, accounting for metal–molecule charge transfer. The same supercell is used as in the contacted molecule junction calculation, but removing the atoms of the leads. The added charge is calculated from a three step procedure:

- the density ρ(*r*) of the complete junction is computed;
- the density ρ′(*r*) of the molecule is also calculated using the same geometry and simulation box as in the junction;
- the added charge is given by integrating ρ(*r*) − ρ′(*r*) over the volume spanned by the molecule. For BDT-h, this volume is given by the region between two planes perpendicular to the S–S axis and passing through the two S atoms (see Figure 6).

The rationale behind the proposed method is to modify the electronic structure of the gas-phase-isolated molecule with the purpose of accounting for the lead–molecule charge transfer. Thus, the isolated molecule is placed into an environment closer to that of the molecular junction. Previous studies [24,26] have already underlined the importance of the lead–molecule charge transfer and the significance of its role in transport properties of molecular junctions. Here, it constitutes the basis for the construction of a new method of analysis.

#### Application of the new method to BDT-h

**Figure 7:**
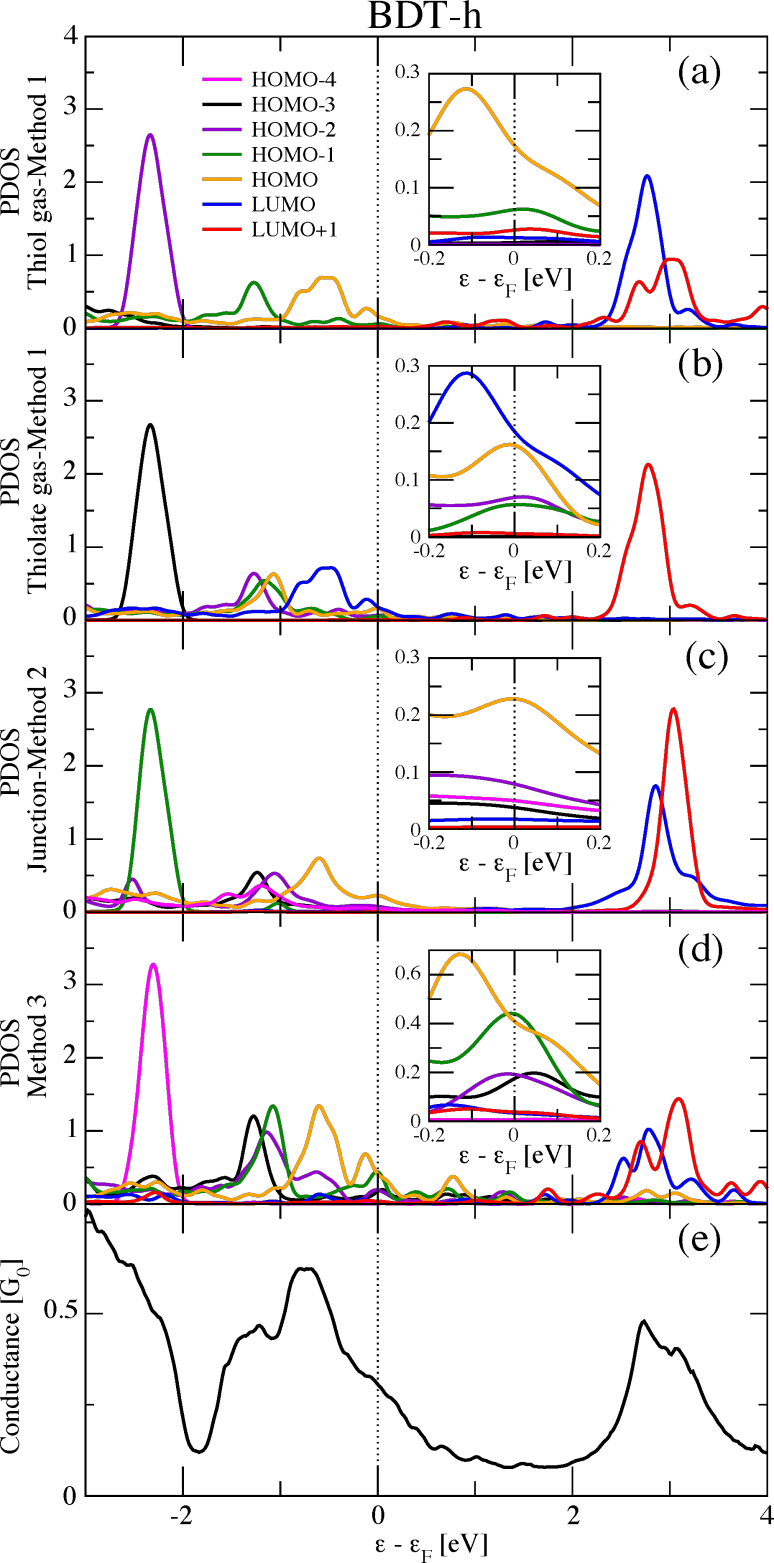
Projected density of states (a,b,c,d) and conductance (e) of benzene-dithiol in the hollow geometry (BDT-h). The PDOS for the different molecular orbitals (from HOMO−3 to LUMO+1) have been obtained with (a) Method 1 based on the dithiol molecule, (b) Method 1 based on the dithiolate molecule, (c) Method 2, and (d) Method 3 (see text). The insets show the details in the Fermi energy region.

We apply our new method to the case of BDT-h (hollow geometry), which presents contradictory results using standard methods, as explained later. According to our recipe, the extra charge to be added to BDT-thiolate to simulate the environment of the BDT-h junction was found to be ≈0.5*e*^−^. However, we observe that the modifications of the MOs are slightly affected by the precise value of the added charge, except when the charge crosses integer values, ρ = 0, 2, …, of the electronic unit charge, *e*^−^, at the onset of the occupation of new levels. The MOs found with this procedure are shown at the bottom of Figure 4. Remarkably, these MOs now look much more similar to the MOs found with Method 2 for BDT-h, as is clearly shown. Furthermore, they present marked differences from the original Method 1 for dithiolate, and in some cases are even closer to Method 1 for the dithiol molecule. Figure 7 shows the PDOS for the BDT-h junction calculated with the traditional methods (Method 1 for dithiol and dithiolate isolated molecules and Method 2 for a selected set of MLWFs for the BDT-h junction) and our proposed Method 3. We focus on the PDOS around ε = 3 eV, where traditional methods present the most important differences. In that energy region, Method 3 provides an obvious improvement. Coming from a dithiolate-isolated molecule, the PDOS from Method 3 is closer to the one from Method 1 for the dithiol molecule than for the dithiolate molecule, thus bridging the gap between the dithiol and dithiolate molecules. Moreover, in this same energy region, when considering the relative height between the PDOS peaks of LUMO and LUMO+1, Method 3 evidently bridges the gap between Method 1 for the dithiol and dithiolate molecules and Method 2. We can probably conclude the same also for the ε = 0 eV region, though restricting the discussion to the PDOS of the HOMO. One can observe the evolution of the PDOS peak of the HOMO at the Fermi energy from Method 1 for the dithiol molecule, from Method 3 and from Method 2. We can say that Method 3 is somewhat successful in reconciling the traditional Methods 1 and 2. However, we do not notice any other evident improvement. Upon detailed inspection of the ε = 0 eV region (not shown), clear differences among the PDOS can be observed. The MO ordering problem continues persists: the PDOS peak at ≈−2.5 eV from Method 3 is attributed to yet another MO, the HOMO−4. The same ambiguous attribution remains for the PDOS of the intermediate HOMO orbitals.

We have also tested Method 3 on the more complex case of BDT-p. The MOs from Method 3 (not shown) do not resemble those from Method 2, and consequently, no satisfactory results on the PDOS were obtained. BDT-p continues to be an unsatisfactory case also for Method 3. This is likely because the metal–molecule charge transfer is not the only, or the main, parameter affecting the electronic structure of BDT-p. This might be due to a possibly higher metal–molecule coupling and hybridization. In conclusion, Method 3 provides encouraging partially satisfactory results, in particular in reconciling the two traditional methods as in BDT-h. However, this is not the case in general, and not all problems are solved. The metal–molecule charge transfer is not the only mechanism at play. One should probably also take into account the metal–molecule hybridization. This is not an easy task if the purpose is to maintain an isolated molecule.

## Discussion

As discussed in the previous section, Method 3 aims at overcoming the drawbacks related to the identification of MOs using Method 1. Instead, one could have explored the possibility to improve upon Method 2. However, as we argue hereafter, this path appears to us less physically grounded. It actually opens even more fundamental questions on the implicit hypothesis at the basis of the interpretation of the conductance based on the PDOS, and raises further doubts on the validity of the whole procedure.

### Dependence of MOs and PDOS from the choice of Wannier functions basis set

**Figure 8:**
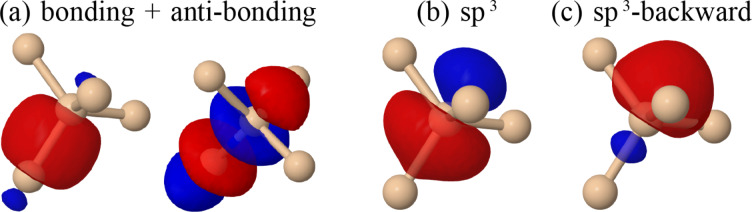
Using the Marzari–Vanderbilt algorithm, three different sets of Wannier functions (WFs) with comparable spread can be obtained for bulk silicon. While (a) bonding + antibonding and (b) sp^3^-forwards are the most “physical” WFs, though not the most localized ones, and (c) sp^3^-backward are the maximally localized WFs.

At first sight, Method 2 (for which MOs originate from the junction) would seem more meaningful for studying the conductance. However, it presents a severe drawback for which it seems very difficult to find a solution. There is a certain arbitrariness in the criterion establishing the spatial limits of a molecule and thus the basis elements that will be considered as being “localized on the molecule”. For instance, there can be MLWFs localized on the molecule–lead bonds as we have pointed out for BDA. It is then quite arbitrary to say whether they are localized on the molecule or on the leads. This choice clearly affects the resulting submatrix, as well as the number and the shape of the MOs found after its diagonalization. Intuitively, these basis elements should have an important effect on the junction conductance, thus it is logical to keep them when generating the MOs. Coming back to the case of BDA, the most important PDOS at the Fermi energy was precisely the one associated to the MO presenting the major localization on the molecule bond MLWF (i.e., the LUMO). If we had discarded the latter from those “localized on the molecule”, we would have excluded this important MO from the analysis of the zero-bias conductance. It is actually reassuring that this MO also appeared when using Method 1, though labeled LUMO+1 due to the already discussed inverted ordering (see Figure 1) and it was also the most important PDOS at ε_F_. However, at the same time, it shows that the exclusion of some MLWFs based on their localization may lead to very different interpretations when starting from Method 1 or Method 2. A strategy to circumvent this drawback is to select a different set of Wannier functions (WFs), or any other localized basis set with elements presenting a well-defined localization (on the molecule or on the leads). For instance, atom-centered basis sets would resolve this ambiguity, such as symmetry-adapted WFs [53], WFs obtained from linear combination of atomic orbitals (LCAO) projections [54], or LCAO basis sets. Furthermore, it is well known that, in some cases, the Marzari–Vanderbilt [42] algorithm can lead to different sets of WFs. For instance, bulk silicon presents at least three different sets of WFs with a similar degree of localization (as measured by the spread, *S*). When starting the Marzari–Vanderbilt algorithm from a random initial guess, there is a high probability to reach the global minimum (*S* = 2.56 Å^2^) for which the lowest eight MLWFs are of the sp^3^-backward type (Figure 8c), which do not correspond to the real chemical orbitals. It is obviously possible to obtain the eight sp^3^-forward WFs (Figure 8b), which correspond to the physical chemical sp^3^ orbitals, but at slightly higher local minimum (*S* = 2.95 Å^2^). Finally, the set of WFs with four bonding orbitals on one Si atom and four antibonding orbitals on the other atom (Figure 8a) has a relatively large spread (*S* = 5.09 Å^2^). However, when performing the search of the MLWFs for the four valence states only, the minimum spread is obtained for a set containing the four bonding orbitals.

The previous discussion highlights a possible ambiguity in Method 2 for identifying the MOs, and hence, in using the corresponding PDOS to interpret the conductance. For a single junction, one may find several sets of WFs. The one presenting the minimum spread (the most localized) does not necessarily correspond to the real physical situation, and this cannot be known a priori. The calculated conductance does not depend on the chosen basis set, provided the basis is complete and at convergence. On the other hand, the submatrix of the junction Hamiltonian does depend on the chosen basis set and so do its eigenfunctions (which define the MOs) and the resultant PDOS. Consequently, the physical interpretation of the conductance by the PDOS does depend on the chosen WF or other basis set. A basis-dependent interpretation method is questionable. Starting from this point, we are led to ask even more fundamental questions: Is the conductance really related to a MO, or a PDOS, or to some MOs and a total PDOS? Before answering these questions, let us try to answer a question even further upstream.

### Is the conductance directly related to the full DOS?

The conductance, 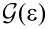, is certainly directly related to the electronic structure of the junction, i.e., to both the electronic energy, ε*_n_*, and wavefunctions, 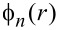, of the extended molecule. Hence, there should also be a relationship to the total DOS, ρ(ε), though somehow indirect and not in a one-to-one correspondence. For instance, wherever ρ(ε) = 0 (no states available at that energy), the conductance 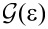 must be also zero. The reverse is not true: the conductance can be zero at energies where the total DOS is finite. This can happen at energies associated with strongly localized wavefunctions with zero spatial overlap among them, for example, core states. There can also be other factors beyond localization, altering the direct relationship between 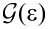 and ρ(ε). For instance, not all delocalized wavefunctions are good conducting channels [55]. As a result, direct conclusions cannot be drawn from the inspection of the DOS only.

### Is the conductance related to some kind of PDOS?

Whether the conductance is directly related to some kind of PDOS, be it onto a given MO or onto some MOs or even the total PDOS, is actually less obvious to answer than for the full DOS. Additionally, this is also true for the physical interpretation of the conductance based on such quantities. Taking the example of BDT-h (Figure 7), one can see that the conductance profile is qualitatively related to a total PDOS including the MOs that are close to the Fermi energy. Nevertheless, it is not possible to observe a quantitative relationship between the conductance value and the total PDOS height. This is more evident in the case of BDT-p (Figure 5), where one cannot explain why the conductance is larger at −1 eV than at 3.5 eV. At below −1 eV, the agreement becomes worse, even qualitatively. In the case of BDA (see Figure 2), the relationship between the conductance and the total PDOS is even less evident. This work has made it clear that the conductance analysis depends on a suitable choice of the MOs. For this reason, the interpretation of the conductance in terms of the PDOS is quite questionable. We should first provide an answer to the following fundamental questions:

- Which set of MOs physically represent the molecule in the junction?
- Given the lead–molecule hybridization, are the MOs obtained from an isolated molecule (i.e., from Methods 1 or 3) meaningful for analysis of a metal–molecule junction?
- Are MOs obtained by diagonalizing a submatrix of the Hamiltonian (Method 2) physical, given the fact that they depend on the choice of basis set?

MOs identified as the eigenvectors of the gas phase, isolated Hamiltonian (Methods 1 and 3) have a physical meaning. However, this is only true for the isolated molecule and not necessarily for the junction. For the latter, the eigenfunctions of the isolated molecule are but another basis set (just like the atomic orbitals for a solid). Furthermore, the actual choice of the molecule may not be unique (e.g., dithiol or dithiolate). As for Method 2, an interpretation which depends on the chosen basis set (e.g., WFs, LCAO, Gaussians or wavelets) cannot be considered physical. We believe that a completely different direction should be taken in order to provide an answer to these questions. What matters for a physical interpretation of the conductance is the full electronic structure of the extended molecule (containing also some layers of the leads). Considering the extended molecule system needed to converge the conductance, which typically contains on the order of 10^2^ gold and 10^1^ molecule atoms, one can realize that the molecule does not even have such an important weight on the determination of the electronic structure of the junction. Following these arguments, we can justify that a meaningful procedure to provide a physical interpretation of a junction conductance should rely on the wavefunctions and energies directly identified for the extended molecule electronic structure. Thus, in order to provide a physical interpretation of the conductance, we believe that the LDOS, a quantity independent from the basis set and directly built on the extended molecule wavefunctions and energies, is the most meaningful. In this sense, we have already presented an application which uses the LDOS for the interpretation of the quantum transport conductance [23]. Regarding an interpretation of the molecular junction conductance rooted in the molecular PDOS, this work first attempted to reconcile the two traditional methods (Methods 1 and 2) by introducing a new one (Method 3). Some success was achieved in this direction, but we cannot consider the problem to be solved. Further work is clearly needed. However, our considerations led us to doubt that a fully satisfactory solution exists along this direction.

## Conclusion

Taking as examples two reference molecular junctions (benzene-diamine and benzene-dithiol between gold leads), we have investigated the interpretation of the conductance based on the PDOS onto molecular orbitals. This is usually identified using one of two procedures: diagonalization of Hamiltonian of the gas phase, isolated molecule (Method 1), or diagonalization of a submatrix of the junction Hamiltonian constructed by considering only basis elements localized on the molecule (Method 2). We have shown that these two methods can lead to substantially different MOs and hence PDOS. Furthermore, within Method 1, the PDOS depends on the isolated molecule chosen to represent the junction (e.g., with or without dangling bonds) and within Method 2, the PDOS depends on the chosen basis set. As a consequence, the analysis of the conductance based on the PDOS can lead to different, if not contrasting, conclusions. This is particularly true for the analysis of the zero-bias conductance, which can be found to be driven by, e.g., the LUMO in one method and the HOMO in another. To counter these drawbacks, we proposed an alternative method (Method 3) as an improvement to Method 1. This new method somewhat reconciles Methods 1 and 2, but still presents problems that point to more fundamental questions. An analysis of the conductance based on the PDOS seems not to rely on well-established roots due to the arbitrariness in the identification of MOs. Our proposed method provided some indications toward possible solutions to the problem of interpreting the molecular junction conductance.

## Acknowledgements

We thank Pierre Darancet, Jeff Neaton and Xavier Blase for useful discussions. GMR acknowledges the F.R.S.-FNRS for financial support. Computational resources were provided by the supercomputing facilities of the Université catholique de Louvain (CISM/UCL), by the Consortium des Équipements de Calcul Intensif en Fédération Wallonie Bruxelles (CÉCI), and by the French GENCI supercomputing center (Project i2012096-655).
